# A drug candidate for a rare polyalanine disease targeting the protein quality control

**DOI:** 10.1016/j.omtn.2024.102364

**Published:** 2024-10-30

**Authors:** Sharon Pochtar, Avraham Ashkenazi

**Affiliations:** 1 The Department of Cell and Developmental Biology, Faculty of Medical and Health Sciences, Tel Aviv University, Tel Aviv 6997801, Israel; 2 Sagol School of Neuroscience, Tel Aviv University, Tel Aviv 6997801, Israel

## Main text

Congenital central hypoventilation syndrome (CCHS) is a rare genetic disease characterized by a dysfunctional ventilatory response to hypercarbia (elevated carbon dioxide levels) and hypoxemia (low oxygen levels), particularly during sleep.^1^ This life-threatening condition results from in-frame triplet duplications in the paired-like homeobox 2B (*PHOX2B*) gene, leading to an expansion of the normal polyalanine (polyAla) stretch from 20 residues to +4 to +13 additional alanine residues.^2^ Longer polyAla expansions correlate with a more severe clinical phenotype. The *PHOX2B* gene encodes a transcription factor critical for developing neurons that regulate autonomic reflexes, including respiratory, cardiovascular, and digestive functions.^3^

The polyAla expansion mutations in PHOX2B impair its ability to properly localize within the nucleus, leading to protein aggregation and cytoplasmic retention, which can perturb cellular homeostasis, further contributing to the accumulation of misfolded PHOX2B proteins.^4^ This aberrant mislocalization also disrupts the transcription factor’s regulation of key target genes, such as dopamine beta-hydroxylase (DBH), which is involved in noradrenergic signaling.^5^ Although compounds like geldanamycin (GA) and its analog, 17-allylaminogeldanamycin (17AAG), have been shown to reduce PHOX2B aggregation and restore nuclear localization, their toxicity limits their potential for clinical use.

In a recent study published in *Molecular Therapy Nucleic Acids*, Africano et al.^6^ utilized RNA sequencing (RNA-seq) and connectivity mapping (CMap) to identify safer therapeutic candidates. These compounds were tested in cells to correct PHOX2B mislocalization and gene regulation. The ability to restore normal respiratory function was tested in Phox2b27Ala/+ mutant mice, a model carrying a heterozygous polyAla expansion that mimics the hypoventilation phenotype seen in patients with CCHS. This study highlights promising new avenues for the treatment development of CCHS and emphasizes the importance of genetic and pharmacological screening techniques.

The study showed that 17AAG treatment restores normal gene expression in PHOX2B+13Ala mutant cells, which are associated with CCHS. Initially, several genes were dysregulated in these mutant cells. However, after treatment, gene expression largely resembled the wild-type profile, with 17AAG affecting various pathways, including protein folding and autophagy, helping to reverse the effects of the polyAla mutation. Due to the toxicity of the 17AAG, the CMap database was utilized to identify bioactive compounds with a similar gene expression signature but lower toxicity. Four clinically relevant compounds—parthenolide, trichostatin A, guggulsterone, and vorinostat (SAHA)—were selected for further testing as potential alternatives to 17AAG for treating CCHS. All four compounds increased the nuclear re-localization of the PHOX2B+13Ala mutant proteins in transfected cells. Although parthenolide showed the best re-localization effect at high doses, it exhibited high cellular toxicity. In contrast, SAHA was the most effective at lower doses for restoring PHOX2B+13Ala transcriptional function and relocating PHOX2B to the nucleus, suggesting it is a promising candidate for further testing.

The study also found that SAHA effectively promotes nuclear re-localization and restores the transcriptional function of PHOX2B proteins with different lengths of polyAla, including the +7Ala expansion mutation that appears to be common in patients with CCHS. Furthermore, SAHA treatment significantly improved breathing-related activity in isolated brainstem preparations from Phox2b27Ala/+ mutant mice, an *ex vivo* model for CCHS. After long-term exposure to SAHA, the mutant brainstem preparations showed increased spontaneous respiratory rhythm and restored sensitivity to acidosis, comparable to wild-type levels. These findings suggest that SAHA can effectively compensate for respiratory deficits associated with CCHS.

**Figure 1:**
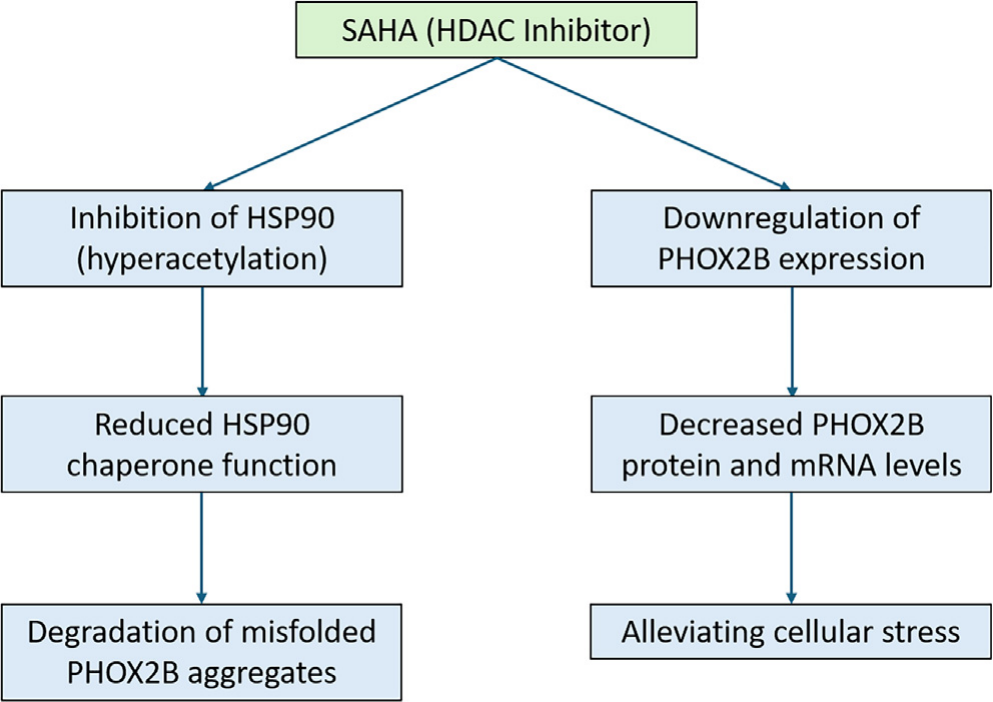
Potential mode of action of vorinostat (SAHA) on protein quality control mechanisms targeting mutant PHOX2B in cell and mouse models of CCHS

As a histone deacetylase (HDAC) inhibitor, SAHA likely operates through two potential pathways (Figure 1). First, by inhibiting the molecular chaperone heat shock protein 90 (HSP90) through hyperacetylation, SAHA reduces its chaperone function, leading to the degradation of client proteins, including mutant PHOX2B aggregates. This effect aligns with the restoration of respiratory parameters observed in *ex vivo* brainstem preparations from Phox2b27Ala/+ mutant mice. Second, SAHA may downregulate PHOX2B expression, lowering mutant PHOX2B protein and mRNA levels to decrease cellular toxicity caused by the polyAla expansion mutations.

Looking ahead, several questions emerge from this study, including whether the use of cell lines and brainstem preparations fully reflects the complex interactions of the central and peripheral autonomic nervous systems, where PHOX2B is expressed. Moreover, because of the restricted postnatal survival of CCHS mutant animals**,** the study was limited to experiments using late embryonic brainstem preparations rather than postnatal or adult models. This constraint may limit the ability to predict the effects of SAHA on long-term respiratory function, which is critical in CCHS. Finally, HSP90 inhibition by SAHA affects multiple client proteins, and therefore the potential impact on other cellular processes needs to be determined. It is clear that assessing the full therapeutic potential of this promising drug warrants further investigation.

## Author contributions

S.P. and A.A. wrote the manuscript and created the artwork.

## Declaration of interests

The authors declare no competing interests.
